# Inflammatory versus Anti-Inflammatory Profiles in Major Depressive Disorders—The Role of IL-17, IL-21, IL-23, IL-35 and Foxp3

**DOI:** 10.3390/jpm11020066

**Published:** 2021-01-23

**Authors:** Małgorzata Gałecka, Katarzyna Bliźniewska-Kowalska, Agata Orzechowska, Janusz Szemraj, Michael Maes, Michael Berk, Kuan-Pin Su, Piotr Gałecki

**Affiliations:** 1 Department of Psychotherapy, Medical University of Lodz, 91-229 Lodz, Poland; 2 Department of Adult Psychiatry, Medical University of Lodz, 91-229 Lodz, Poland; katarzyna.blizniewska-kowalska@umed.lodz.pl (K.B.-K.); agata.orzechowska@umed.lodz.pl (A.O.); piotr.galecki@umed.lodz.pl (P.G.); 3 Department of Medical Biochemistry, Medical University of Lodz, 92-215 Lodz, Poland; janusz.szemraj@umed.lodz.pl; 4 Department of Psychiatry, Faculty of Medicine, Chulalongkorn University, Bangkok 10330, Thailand; dr.michaelmaes@hotmail.com; 5 IMPACT—The Institute for Mental and Physical Health and Clinical Translation, School of Medicine, Barwon Health, Deakin University, Geelong, VIC 3220, Australia; michael.berk@deakin.edu.au; 6 Orygen, The National Centre of Excellence in Youth Mental Health, 35 Poplar Road, Parkville, VIC 3052, Australia; 7 The Department of Psychiatry and The Florey Institute of Neuroscience and Mental Health, The University of Melbourne, Parkville, VIC 3010, Australia; 8 An-Nan Hospital, China Medical University, Tainan 709, Taiwan; cobol@cmu.edu.tw

**Keywords:** depression, MDD, autoimmunity, inflammation, interleukin 17 (IL-17), interleukin 21 (IL-21), interleukin 23 (IL-23), interleukin 35 (IL-35), Foxp3, psychiatry, mental disorders, neuroscience, psychiatry

## Abstract

Background: The authors of this research study intended to verify whether there are any changes in gene expression in depressed patients without coexisting inflammatory diseases for selected immune-inflammatory factors that are particularly important in autoimmune disease pathogenesis (IL-17, IL-21, IL-23, IL-35, Foxp3). Methods: The study was carried out on a group of 190 patients with depression and 100 healthy volunteers. The severity of depressive symptoms was assessed using the Hamilton Depression Scale. RT-PCR was used to evaluate mRNA expression and ELISA was used to measure protein expression of these genes. Results: The level of gene expression for IL-17, IL-21, IL-23, and IL-35 was substantially higher in the group of patients with depression compared to the control group. The mean mRNA expression of Foxp3 was considerably reduced in patients suffering from depressive disorders. There was a statistically significant correlation between the number of hospitalizations and the expression of specific inflammatory factors. Conclusions: Expression of specific inflammatory genes may be a factor in the etiopathogenesis of depressive disorders. The duration of the disease seems to be more important for the expression of the genes in question than the severity of depression. These cytokines may affect the metabolism of neurotransmitters and neuroendocrine functions in the brain as well as be a marker and a new potential therapeutic target for recurrent depressive disorders.

## 1. Introduction

Severe depression is a common and serious disorder of a recurrent nature that is linked with the reduction of vital functions. Furthermore, it negatively affects the quality of life, morbidity, and mortality [1,2,3,4,5]. The risk of major depression development during a lifetime is estimated at 8–12% [6]. Undoubtedly, the prevalence of depression is increasing, which is associated with working ability limitation and the risk of premature death. Approximately 20 to 50% of those suffering from depression commit suicide [7].

The last two decades of research confirm the significant role the immune system plays in the process of depression development. The comorbidity of depressive disorders with autoimmune disorders is firmly established. Somatic diseases can be exacerbated by a comorbid depressive state and may lead to a worse scenario and cause the disease to be longer [8]. In addition, specific neuropsychiatric presentations, for example psychomotor retardation and fatigue, can lead to diagnostic overlap and poorer response to therapy. Depressive disorders often precede the development of autoimmune diseases. Depression is a predictor of the development of rheumatoid arthritis and increases the risk of its occurrence by 38% [9]. Overlapping pathophysiology is confirmed by various research studies which found that perinatal depression significantly more often affects people with a diagnosis of multiple sclerosis (MS). Importantly, more than 50% of individuals suffering from perinatal depression with diagnosed MS, manifested earlier depression, and their children had a 34% higher rate of mental disorder compared to children without parental MS. Additionally, the incidence of mental disorders was higher among the children whose parents struggled with perinatal depression in comparison to the children whose parents did not have any episodes of perinatal depression. Moreover, parental MS and perinatal depression result in an increase in the risk of mental disorder development in children [10]. The underlying pathophysiology is complex, associated with the severity of inflammatory reactions in both the central and peripheral nervous system [11,12,13].

Considering the role of inflammatory processes in depression, the study aimed to verify the association of altered inflammatory processes (altered peripheral gene expression of inflammatory mediators) with major depressive disorders (MDD) by measuring changes in the levels of inflammatory indicators (IL-17, IL-21, IL-23, IL-35 and Foxp3), important in various types of autoimmune diseases, like rheumatoid arthritis (RA) and multiple sclerosis (MS).

## 2. Materials and Methods

### 2.1. Participants

The research was carried out on a group of 290 individuals. As many as 190 people diagnosed with recurrent depressive disorders were eligible to take part in the study. These participants were aged 20 to 67 (mean age M = 47.51, SD = 11.18) and included 117 women (61.6%) and 73 men (38.4%). The diagnostic criteria for recurrent depressive disorders and depressive episodes based on the ICD-10 classification (F32.0–F32.2, F33.0–F33.8) were accepted as inclusion criteria in the study [14]. The control group comprised 100 healthy volunteers with no history of psychiatric immune or other major medical disorders, 65 of whom were women and 35 men. The mean age of the participants was 41.29 ± 13.50 years.

### 2.2. Safety and Exclusion Criteria

All the participants were recruited during hospital treatment at the Medical University of Lodz, Poland. The experiment did not affect background pharmacotherapy and psychotherapy. Exclusion criteria included a diagnosis of another mental disorder or past somatic diseases which may have had a significant impact on the course of a depressive episode, as well as coexistence of inflammatory, autoimmune, or neoplastic diseases assessed at interview. Each participant provided written consent to participate in the study in compliance with a report approved by the Bioethics Committee of the Medical University of Lodz (approval No RNN/883/11/KB of 13/12/2018).

### 2.3. Method for Assessing Depression Severity

The severity of depression symptoms was evaluated and assessed using the Hamilton Depression Rating Scale (HDRS, HAM-D). With regard to this scale, Cronbach’s alpha totaled 0.70; the sensitivity coefficient was 0.78, and the test relevance coefficient reached 0.75 [15].

### 2.4. Method for Assessing Biological Parameters

#### 2.4.1. Protein Concentration

An analytical curve for serum albumin was determined and the total protein concentration in the serum samples of the patients was measured. The analyzed samples and reference samples were carried out parallelly in three repetitions. Multiskan Ascent Microplate Photometer (Thermo Labsystems, Philadelphia, PA, USA) was used to quantify sample absorbance at λ = 562 nm; additionally, the standard curve equation made it possible to calculate total protein concentration.

#### 2.4.2. Enzyme-Linked Immunosorbent Assay (ELISA)

Serum concentration of IL-17, IL-21, IL-23, IL-35, and Foxp3 *proteins* was measured according to the manufacturers’ protocols with the use of Human IL-17 Quantikine ELISA Kit, Human IL-23 Quantikine ELISA Kit (R&D Systems, Inc. Minneapolis, MN, USA), Human IL21 ELISA Kit (CLIA) from LifeSpan BioSciences (Biocompare South San Francisco, CA, USA), Human IL-35 ELISA Kit and Human FOXP3 ELISA Kit (MyBioSource, Inc., San Diego, CA, USA). An endogenous control of protein concentration in the samples collected from the patients was performed with β-actin. Human Actin Beta (ACTb) ELISA Kit (BMASSAY) was used in this case in line with the recommendations specified by manufacturer. Multiskan Ascent Microplate Photometer (Thermo Labsystems) allowed the absorbance of the samples to be measured and analyzed at λ = 450 nm. Analytical curves for the proteins were prepared with the aim of determining protein concentration.

#### 2.4.3. Isolation of Total RNA

InviTrap Spin Universal RNA Kit (Stratec molecular, Berlin, Germany) enabled total RNA isolation to be conducted from the patients’ peripheral blood lymphocytes, this process was consistent with the recommendations of the manufacturer. A spectrophotometer (Picodrop -VWR International Corporate LLC ) made it possible for the authors to measure absorbance values at λ = 260 nm with an intention to verify total RNA concentration. Isolated RNA was stored at a temperature of −70 °C.

#### 2.4.4. Isolated RNA Quality Analysis

Total RNA quality was established using Agilent RNA 6000 Nano Kit (Agilent Technologies–Santa Clara, CA, USA) and based on the protocol prepared by the manufacturer. RNA 6000 Nano dye (1 µL) was added to a test tube which already contained 65 µL of Agilent RNA 6000 Nano gel matrix. The test tube was then centrifuged (10 min, 13,000× *g*). The authors applied the gel-fluorescent dye mixture onto the surface of a Nano chip placed in a workstation. Afterwards, RNA Nano marker (5 µL) was added to selected pits. Denaturation of the RNA and RNA size marker isolated samples was performed for 2 min at a temperature of 70 °C. Following, 1 µL of the sample was added to selected pits of the Nano chip and mixed (1 minute, 2400 rpm). The authors verified and measured isolated RNA quality with special equipment (specifically 2100 Bioanalyzer, Agilent Technologies). Total RNA degradation was calculated on the bases of electrophoretogram and specific RIN values were documented. A further analysis of the samples was performed only when the RIN value was > 7.

#### 2.4.5. RT-PCR Reverse Transcription

TaqMan^®^ RNA Reverse Transcription Kit (Applied Biosystems–Foster City, CA, USA) was used by the authors of the study to carry out a Reverse Transcription (RT) reaction. The entire process was based on the recommendations and protocols specified by the manufacturer, and the following probes, supplied by Applied Biosystems, respectively, for IL-17, IL-21, IL-23, IL-35, Foxp3, and RPL13A, were used: Hs00174383_m1, Hs00222327_m1, Hs00372324_m1, Hs04931857_m1, Hs01085834_m1, Hs04194366_g1. Incubation of the samples (30 min, 16 °C and 30 min, 42 °C) took place in a thermocycler (Biometra). Reverse transcriptase was inactivated for 5 min at 85 °C and the cDNA obtained was stored at −20 °C.

#### 2.4.6. Real-Time PCR Reaction

Following recommendations of the manufacturer, a real-time PCR reaction was carried out using TaqMan^®^ Universal PCR Master Mix, No UNG (Applied Biosystems). The Ct comparative method was used by the authors to calculate miRNA relative expression of the analyzed genes [16]. Gene expression levels for IL-17, IL-21, IL-23, IL-35, and Foxp3 in specific tissues were normalized with respect to the reference *gene* (RPL13A).

A separate 96-well plate was used to amplify each target probe. Sample incubation was carried out at a temperature of 50 °C for 2 min and at 95 °C for 10 min. After that, the samples were cycled for 30 s at 95 °C, for 30 s at 60 °C and for 1 min at 72 °C. In total, the authors performed 40 cycles. Data regarding fluorescence emission was recorded and mRNA levels were computed using the critical threshold (Ct) value. ABI Prism 7900 HT (SDS Software – Applied Biosystems, Foster City, CA, USA) enabled the analyses and calculations to be carried out. Controls without RT and with no template cDNA were completed with each assay. ∆∆Ct standard 2^−∆∆ct^ calculations enabled relative gene expression—expressed as a fold change of the control sample—to be determined [17].

### 2.5. Statistical Analysis

The authors performed a statistical analysis of the results in STATISTICA 12.0 PL (Stat Soft Poland, Cracow, Poland. A two-tailed critical area was assumed when the hypotheses were subject to a statistical verification. The types of measurement were chosen following an analysis of the examined variables, which showed that there were no grounds for rejecting the null hypothesis concerning normal distribution. The statistical significance of the relationship between the analyzed variables among the patients treated for depressive disorders was revealed following a statistical analysis based on parametric tests. The materiality level totaled *p* < 0.05 in all the statistical methods used. To assess the relationship between the biomarkers and the number of hospitalizations, the number of depressive episodes, disease duration, HDRS score, and Spearman’s rank order correlation coefficients were used. Due to the ordinal nature of these variables and the non-linear relationship between them, the significance was set at α = 0.05 [18].

## 3. Results

**Figure 1:**
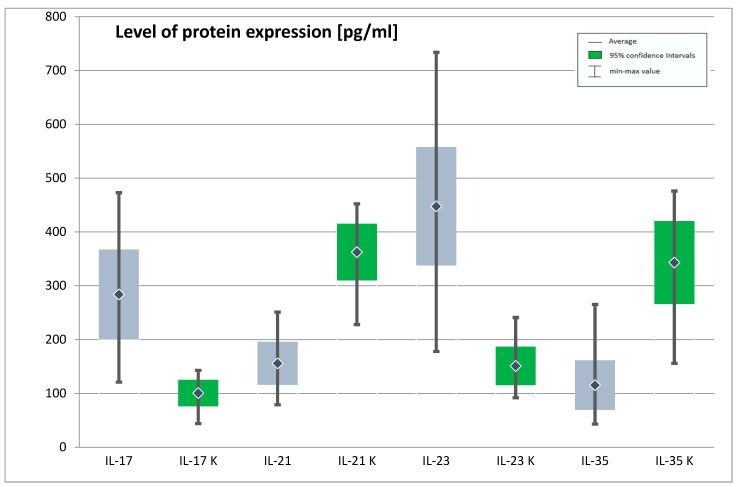
Expression of genes IL-17, IL-21, IL-23, IL-35 at protein level in the study and control group (IL-17—Interleukin 17, IL-21—interleukin 21, IL-23—interleukin 23, IL-35—interleukin 35, K—control group). The figure shows the arithmetic mean, minimum and maximum values of gene expression with confidence intervals of 95%.

A difference between the mean protein expression of IL-17, IL-21, IL-23, and IL-35 in the group of patients with depression and in the control group was statistically significant. The mean protein expression of IL-17 and IL-23 was significantly higher in the depressed patients than in the control group, whereas IL-21 and IL-35 protein expression was significantly lower in depressed patients. In case of protein expression for Foxp3, a difference between the groups that would be statistically significant was not confirmed (Table 1, Figure 1).

**Figure 2:**
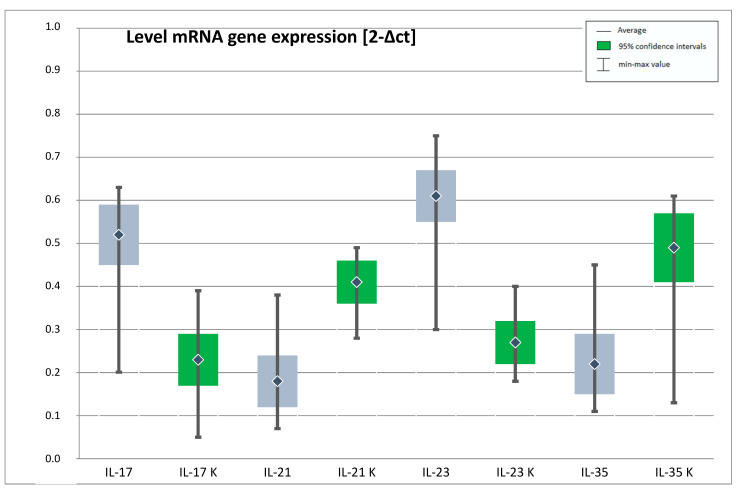
Expression of *genes* IL-17, IL-21, IL-23, IL-35 at the mRNA level in the study and control group (IL-17—interleukin 17, IL-21—interleukin 21, IL-23—interleukin 23, IL-35—interleukin 35, K- control group). The figure shows the arithmetic mean, minimum and maximum values of gene expression with confidence intervals of 95%.

The average mRNA gene expression for IL-17, IL-23 was substantially higher in the group of individuals with depression in relation to the control group. However, the mean expression for IL-21, IL-35 and of the Foxp3 gene at mRNA level was significantly reduced in the patients with depressive disorders. (Table 2, Figure 2).

There were no significant sex-related differences in mRNA and protein expression for IL-17, IL-21, IL-23, IL-35, and Foxp3. The analysis did not confirm any statistical significance for the whole population as well as for the depressed patients and the healthy participants. On the other hand, age was statistically significant regarding the variables studied only in the control group and for the whole population (Table 3).

A positive and statistically significant correlation between the age of the patients and the expression of IL-17 at protein and mRNA levels was found both in the healthy participants (control group) and in the whole population of the experiment, such that the older the age of the patients, the higher the expression of the interleukins. There was also a considerable negative relationship between the age of the participants and the expression of IL-21 at protein and at mRNA levels in the control group, as well as the protein expression of this interleukin in the whole population. Considering all participants, there were also statistically significant correlations between age and the expression of IL-23 at protein level (*r* = 0.5677, *p* < 0.05) and at mRNA level (*r* = 0.6257, *p* < 0.05), between age and the expression of IL-35 at protein level (*r* = -0.5420, *p* < 0.05) and at mRNA level (*r* = −0.5313, *p* < 0.05), and between age and the expression of the Foxp3 gene at protein level (*r* = −0.5776, *p* < 0.05) and at mRNA level (*r* = −0.5863, *p* < 0.05).

The experiment also analyzed the correlation between the expression of the analyzed genes and clinical variables, such as the number of psychiatric hospitalizations, disease duration, the number of depressive episodes, and the HDRS score. There was a statistically significant positive link between the number of hospitalizations and mRNA expression of IL-23 (*R* Spearman = 0.16, *N* = 186, *t* = 2.25, *p* = 0.025) and statistically significant negative correlation between the number of hospitalizations and the expression of the Foxp3 gene at both mRNA and protein levels (*R* Spearman= −0.15, *N* = 186, *t* = −2.10, *p* = 0.037 and *t* = −2.07, *p* = 0.040, respectively). The expression of other interleukins did not correlate significantly with the number of hospitalizations.

A statistically significant negative correlation was demonstrated between the expression of the Foxp3 gene (both at protein and mRNA levels) and disease duration (*r* = −0.20, *r* = −0.17, respectively), and also between the expression of this gene and the number of disease episodes (*r* = −0.16, *r* = −0.15, respectively).

No correlation that would be considered statistically significant (*p* > 0.05) was observed between the number of depressive episodes, disease duration and expression with any of the interleukins studied.

The severity of depressive symptoms, as measured with the HDRS scale, did not correlate significantly with any of the variables studied.

## 4. Discussion

Numerous scientific reports indicate the coexistence of depressive disorders with autoimmune diseases [19]. In our experiment, we confirmed the importance of gene expression of selected inflammatory indicators for depression. Specifically, concentrations of IL-17, IL-21, IL-23, and IL-35 were significantly higher in the depressed group. The expression of the Foxp3 gene appeared to decrease with the progression of the disease. It seems that the duration of the disease is more important for the expression of the examined genes than severity.

### 4.1. Interleukin 17 (IL-17)

The gene for interleukin 17 (IL-17) was among the factors analyzed during the experiment. It is a cytokine of proven importance in the pathogenesis of, among others, psoriasis and rheumatoid arthritis [20,21]. This interleukin, and in particular its main subtype IL-17A, is produced by a special subpopulation of T lymphocytes, i.e., the so-called Th17 cells. Secukinumab is a monoclonal antibody that binds to interleukin 17A and, thus, inhibits its interaction with a receptor on the cell surface. It is used in the immunotherapy of psoriasis or rheumatoid arthritis (RA).As a consequence, secukinumab inhibits proinflammatory cytokines release and reduces the role of IL-17 in causing the symptoms of the previously mentioned autoimmune diseases. Interestingly, an improvement in depressive symptoms was observed in some patients who used this immunotherapy [22]. This may be related to the general improvement of the clinical condition and reduction of symptoms in this group of patients, but also to the possible common inflammatory background of depression and autoimmune diseases [23]. In a cohort study carried out by Kurd et al., the prevalence of depression, anxiety and suicidal ideation in psoriasis patients is 25.9, 20.9 and 0.9 per 1000 people per annum, respectively. Furthermore, patients suffering from a severe form of psoriasis showed a 72% increase in the risk of depression development compared to the control population [24]. In a case series of patients with psoriasis and concomitant mental disorders by Esposito et al., a good long-term efficacy and safety profile for secukinumab treatment was demonstrated, despite concomitant psychiatric therapies that are known to aggravate psoriasis, such as sodium valproate or lithium [25]. An improvement in mental health was also observed [26]. Interleukin-17 also plays an important role in rheumatoid arthritis pathogenesis. Albeltagy et al. [27] examined 120 patients suffering from rheumatoid arthritis, of whom 2/3 presented depressive symptoms (BDI II 23.95 ± 8.17). Depressive symptoms were strongly associated with increased levels of, among others, IL-17 and IL-6 as well as with rheumatic disease activity [27]. A relationship between mood disorders and exacerbation of RA was also observed by Li et al. (2019).They examined 113 patients with RA and 42 healthy volunteers and evaluated them for anxiety and depression symptoms using the HADS scale. The severity of RA was assessed using the disease activity score DAS28. Serum levels of proinflammatory cytokines, including interleukin 6 and 17, were measured and compared between different patient groups depending on disease activity and pain level. They showed that the higher the IL-6 and IL-17, the higher the severity of RA and the severity of depressive symptoms [28]. A positive feedback loop is observed between IL-6 and IL-17 [29]. The link between autoimmune diseases and depression, the research of which has been presented above, is scientifically well established. Research also indicates and confirms pro-inflammatory activation for Th17 in the people with depressive disorders. Owing to the fact that there are not many studies conducted on patients with depressive disorders and IL-17 expression, it is difficult to draw a clear conclusion. The subject matter requires more research and analysis, as well as a deeper understanding of the mechanism of action of Th 17 cells in depressive disorders. The role of Th17 cells is a relatively recently discovered area of research regarding depression. It is the result of a growing number of studies related to the occurrence of inflammation in depression. These studies concern—to a large extent—IL-1β, IL-6 and TNFα whose elevated levels in blood occur in patients with depression and in situations of mobilization due to chronic stress, anxiety and inflammatory diseases. Moreover, their presence is an essential element for cell differentiation towards Th17 [30]. Studies carried out by two teams have confirmed, as have ours, an increase in the level of Th17 cells in blood in depressed patients [31,32]. The hypothesis about the influence of depression on the promotion of cells towards Th17 is also confirmed by studies on the impact of antidepressants on the balance of the ratio between pro-inflammatory Th17 cells and anti-inflammatory regulatory T lymphocytes (Treg) [33]. There are also data which show that IL-17A levels do not always correlate with depression [34,35]. The current state of knowledge, as well as the research we have conducted, allows us to conclude that the IL-17 produced by Th17 cells is important in the development of depressive disorders. Further research into the mechanism of Th17 in depression is required to determine the direction of the impact. However, our study shows a relationship between disease duration and the number of hospitalizations and IL-17 levels, which may suggest the hypothesis that it is chronic depressive disorders that affect cell differentiation towards Th17 and play a significant role in relapse. The question of whether Th17 cells can be a therapeutic target or a biomarker of recurrent depression remains to be examined further.

### 4.2. Interleukin 23 (IL-23)

**Figure 3:**
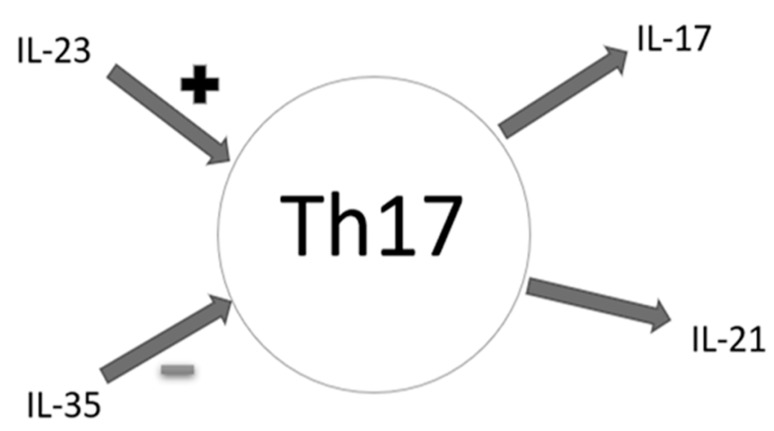
Effects of selected cytokines on Th17 cells—schematic diagram (IL-17—interleukin 17, IL-21—interleukin 21, IL-23—interleukin 23, IL-35—Interleukin 35, ‘+’—promoting effect, ‘-’—inhibitory effect, Th17—Th17 lymphocytes).

There are many interactions between cytokines in the complex human immune system (Figure 3). IL-23 is a key cytokine for the proper functioning of IL-17 producing Th17 cells [36]. Its ability to strongly enhance the expansion of T helper 17 cells (Th17) indicates importance for many inflammatory autoimmune responses [36]. Interleukin 23 belongs to the IL-12 proinflammatory cytokine family [37,38]. It is the main cytokine connecting congenital and adaptive arms of the immune response [39], necessary to induce an early local inflammatory response [40]. Interleukin 23 induces the production of IFN-γ [41], which is important in Th1 mediated responses and cellular immunity against intracellular pathogens. Additionally, IL-23 plays a leading role in NK cell activation, enhancement of T-cell proliferation and regulation of antibody production [41]. Several autoimmune diseases, such as psoriasis, inflammatory bowel disease (IBD), rheumatoid arthritis (RA), and multiple sclerosis (MS), are characterized by elevated amounts of IL-23 [41]. Psoriasis is a disease in which IFN-γ [42] and IL-17 [43] play an important role, hence the significance of IL-23 in the pathogenesis of this disease is not surprising. Interestingly, unlike in our studies on patients with depression, serum IL-23 levels in patients with psoriasis were negatively correlated with disease duration. Interleukin 23 was higher at an earlier stage [36]. Moreover, similarly to psoriasis, a positive correlation was observed in rheumatoid arthritis [RA] between the levels of IL-23 in serum and synovial fluid of patients and the production of other proinflammatory cytokines, i.e., IL-17, IL-1β or TNF-α [36]. An increasing number of targeted biological therapies aimed at IL-23 are being developed. Ustekinumab prevents the integration of IL-12/IL-23 with their receptor, thus blocking later signaling, differentiation and production of the cytokines that are crucial for autoimmune diseases such as psoriasis [44]. Like ustekinumab, briakinumab is a human monoclonal antibody that targets IL-12 and IL-23 through their common subunit p40 [45]. In their study, Langley et al. [46] demonstrated that patients taking ustekinumab for psoriasis showed a significant improvement in depressive symptoms [46]. These observations were confirmed by Kim et al. [34]. Treatment with ustekinumab significantly reduced symptoms of depression (p <0.05) [47]. In our study, we observed a statistically significant increase in IL-17 in peripheral blood and an increase in IL-23 concentration necessary for the stabilization and functioning of this line of lymphocytes. The pathogenesis of many autoimmune diseases was associated for a long time with the IFN-γ releasing population of Th1 cells. Presently, the relationship between Th1 and Th17 is being searched for as both cell populations seem to take an active part in the pathogenesis of auto-aggression processes. Indeed, it has already been proven that Th1 lymphocytes show moderate expression of the IL-17 receptor on their surface, which—acting through this receptor—inhibits the expression of genes of the Th1–T-bet lymphocyte transcription factor, as well as inhibits the production of IFN-γ. This leads to the elimination of the Th1 function. Conversely, in the absence of Th17, the response of Th1 cells is intensified [48]. However, such phenomena do not fully explain the increased concentration of IL-17 and IL-23 in autoimmune diseases. It seems that—in addition to mutual counter-regulation—Th1 and Th17 lymphocytes may need each other, and the cytokines produced and secreted by one cell line initiate the inflammatory process, allowing the influx of another cell population. Detecting increased concentrations of IL-17 and IL-2 in patients with depression can contribute to a better understanding of its pathomechanism.

### 4.3. Interleukin 21 (IL-21)

Another important cytokine secreted by Th17 lymphocytes is IL-21, which acts in an autocrine manner and promotes lymphocyte polarization towards the Th17 phenotype and directly influences the proliferation and maturation of B lymphocytes, CD8 + T cells, and NK cells [49,50]. IL-21 levels are elevated in peripheral blood and tissues of patients with various autoimmune diseases, including rheumatoid arthritis (RA), systemic lupus erythematosus (SLE), immune thrombocytopenic purpura (ITP), type 1 diabetes mellitus (DM 1), autoimmune thyroid disease (AITD), primary Sjogren’s syndrome (pSS), and psoriasis [51]. This interleukin also may have importance in the pathogenesis of depression. Davami et al. [52] found that the level of IL-21—unlike IL-17—did not differ significantly way between depressed patients and healthy individuals (84.30 ± 4.57 vs. 84.12 ± 4.15 pg/mL (*p* > 0.05), respectively) [52]. In our study we observed that protein expression of IL-21 was significantly higher in the individuals with depression.

### 4.4. Interleukin 35 (IL-35)

The main function of IL-35 is to inhibit Th1 and Th17 dependent immune response while supporting regulatory T lymphocytes [53,54,55]. This should suggest that its concentration should be lowered in patients with autoimmune disorders, depending on the Th17 axis. However, our experiment did not confirm this hypothesis. IL-35 expression in patients with depression was higher in a statistically significant way. It unsure whether this was due to the negative feedback loop between IL-35 and Th17 axis cytokines.

### 4.5. Foxp3

The main transcription factor in charge of regulatory T lymphocytes formation is Foxp3 [56]. The central role of regulatory T lymphocytes (Treg) is to control inflammation, which may indicate their deficiency in autoimmune diseases. This is consistent with the results of our experiment, which showed that the average mRNA expression for the Foxp3 gene was significantly reduced in patients with depressive disorders. Foxp3 is a recognized mediator of regulatory T lymphocytes in the peripheral nervous system (PNS) and central nervous system (CNS). The levels of both Foxp3 mRNA and protein reach their highest values during embryonic development and then continue to decrease. In adulthood, the Foxp3 protein concentration, but not mRNA, increases to levels equivalent to the embryonic stage of life. The levels of Foxp3 protein in PNS were low in the embryo, increased throughout life and reached maximum levels in adulthood. The patterns observed for both PNS and CNS were similar in men and women at all stages of development [57]. It is difficult to interpret unambiguously the phenomenon of Foxp3 levels dropping in patients with depression. It may be assumed that immunological changes, i.e., a decrease in anti-inflammatory capacity, occur similarly to the aging process.

Regarding the pro- and anti-inflammatory balance, it is worth mentioning the important role of the Compensatory Immune-Regulatory Reflex System (CIRS) in the pathogenesis of MDD. CIRS is responsible for primary immune-inflammatory response regulation and contributes to spontaneous and antidepressant-promoted recovery from the acute phase of illness. Additionally, signs of activated CIRS pathway can be noticed in patients suffering from depression during remission. This indicates that the original homeostasis after an acute episode cannot be recovered, while subsequent mood disorder episodes are characterized by sensitized CIRS responses. This can explain why the duration of the disease is more important for the expression of the examined genes than its severity [58].

## 5. Conclusions

The experiment shows that IL-17, IL-21, IL-23, IL-35 and Foxp3 concentration can be a marker in the diagnosis of depressive disorders. The mRNA gene expression for IL-17, IL-23 and IL-35 was higher in patients with depression. The analysis presented above displayed a significant positive correlation between the number of hospitalizations and the mRNA expression of IL-23, a negative correlation between the number of hospitalizations and the mRNA expression of IL-35, and a negative correlation between the number of hospitalizations and the expression of the Foxp3 gene at both mRNA and protein levels.

A negative correlation between the expression of the Foxp3 gene and the duration of the disease, as well as between the expression of this gene and the number of disease episodes, was confirmed. This suggests that the expression of the Foxp3 gene decreases with the duration of the disease. Therefore, it can be concluded that the duration of the disease is more important for the expression of the examined genes than its severity. In conclusion, these results may contribute to earlier detection of chronic inflammatory depression and discovering new therapies using biological drugs targeting these pathways.

## Figures and Tables

**Table 1:** Expression of IL-17, IL-21, IL-23, IL-35 genes at protein level in the study and control group

Protein Expression (ng/mL)	Study Group				Control Group				*t*	*p*
	M	Min	Max	SD	M	Min	Max	SD		
**IL-17**	283.73	121.00	473.00	83.39	100.80	44.00	143.00	24.68	21.43	0.000 *
**IL-21**	155.98	79.00	251.00	39.81	362.42	228.00	452.00	52.65	−37.43	0.000 *
**IL-23**	447.66	178.00	734.00	110.08	151.11	92.00	241.00	35.76	26.20	0.000 *
**IL-35**	115.53	43.00	265.00	46.06	342.99	156.00	476.00	77.16	−31.40	0.000 *
**Foxp3**	3.62	1.26	9.63	1.12	7.58	3.34	9.51	1.35	−26.60	0.000 *

(M—arithmetic mean; Min—minimum; Max—maximum; SD—standard deviation; t—Student’s t-test result and is calculated difference represented in units of standard error; p—statistical significance, *- statistically significant; IL-17—interleukin 17, IL-21—interleukin 21, IL-23—interleukin 23, IL-35—interleukin 35, Foxp3—forkhead box P3).

**Table 2:** Expression of IL-17, IL-21, IL-23, IL-35, Foxp3 genes at mRNA level in the study and control group.

mRNA Gene Expression (2−Δct)	Study Group				Control Group				*t*	*p*
	M	Min	Max	SD	M	Min	Max	SD		
**IL-17**	0.52	0.20	0.63	0.07	0.23	0.05	0.39	0.06	33.96	0.000 *
**IL-21**	0.18	0.07	0.38	0.06	0.41	0.28	0.49	0.05	−32.92	0.000 *
**IL-23**	0.61	0.30	0.75	0.06	0.27	0.18	0.40	0.05	45.67	0.000 *
**IL-35**	0.22	0.11	0.45	0.07	0.49	0.13	0.61	0.08	−29.91	0.000 *
**Foxp3**	0.11	0.06	0.18	0.02	0.21	0.12	0.26	0.03	−29.60	0.000 *

(M—arithmetic mean; Min—minimum; Max—maximum; SD—standard deviation; t—Student’s t-test result and is calculated difference represented in units of standard error; p—statistical significance, *-statistically significant; IL-17—interleukin 17, IL-21—interleukin 21, IL-23—interleukin 23, IL-35—interleukin 35, Foxp3—forkhead box P3).

**Table 3:** Correlation of age and expression of genes IL-17, IL-21, IL-23, IL-35, Foxp3 at protein and mRNA levels for the control group and all participants.

**Correlation of Age and Expression of Genes for the Control Group**										
	**IL 17 Protein**	**IL 17 mRNA**	**IL 21 Protein**	**IL 21 mRNA**	**IL 23 Protein**	**IL 23 mRNA**	**IL 35 Protein**	**IL 35 mRNA**	**Foxp3 Protein**	**Foxp3 mRNA**
***r***	0.2437	0.3098	−0.3101	−0.3110	0.0392	0.0282	0.1470	0.1293	−0.0223	−0.0399
***p***	0.015 *	0.002 *	0.002 *	0.002 *	0.700	0.782	0.147	0.202	0.827	0.695
**Correlation of Age and Expression of Genes for All Participants**										
***r***	0.5329	0.6235	−0.6268	0.6118	0.5677	0.6257	−0.5420	−0.5313	−0.5776	−0.5863
***p***	0.000 *	0.000 *	0.000 *	0.000 *	0.000 *	0.000 *	0.000 *	0.000 *	0.000 *	0.000 *

(IL-17—interleukin 17, IL-21—interleukin 21, IL-23—interleukin 23, IL-35—interleukin 35, Foxp3—forkhead box P3); *r*—correlation coefficient; *p*-significance; *-statistically significant.

## Data Availability

Not applicable.
